# Comparison of Iron alone and Zinc Plus Iron Supplementation Effect on the Clinical and Laboratory Features of Children with Iron Deficiency Anemia

**Published:** 2019-10-01

**Authors:** Borhan Moradveisi, Parin Yazdanifard, Nima Naleini, Mohsen Sohrabi

**Affiliations:** 1 Cancer and Immunology Center, Research Institute for Health Development, Kurdistan University of Medical Sciences, Sanandaj, Iran; 2 Pediatric ward, Besat Hospital, Kurdistan University of Medical Sciences, Sanandaj, Iran

**Keywords:** Anemia, Iron-deficiency anemia, Iron, Zinc

## Abstract

**Background:** Childhood Iron deficiency anemia is one of the main health problems around the world especially underdeveloped countries. Supplementation with micronutrients specifically iron supplementation can be considered as a therapeutic strategy to prevent and treatment of this type of anemia. The aim of the present study is to compare the therapeutic effects of zinc plus iron and iron alone supplementation on the clinical and laboratory features of children with iron deficiency anemia referred to our Hospital in 2016.

**Materials and Methods:** 88 patients aged 6 months to 4 years old with iron deficiency anemia and after applying exclusion criteria were enrolled in the study. Patients were randomly divided into two groups to receive zinc plus iron sulfate or iron sulfate alone supplement for one month. After treatment, clinical symptoms and lab test data including CBC, TIBC and serum iron and ferritin levels were again evaluated. Statistical analyses were performed using SPSS15.

**Results:** After one month of treatment, the clinical symptoms relived significantly in both groups. Also, there was significant changes between the mean value of laboratory parameters before and after treatment within each group (P <0.05). However, after one month of treatment there was no significant difference between the two groups (P> 0.05).

**Conclusion:** The study revealed both iron alone and zinc plus iron supplementation are effective on the treatment of iron deficiency anemia but there are no significant difference and preference between these two types of treatment.

## Introduction

 Anemia is defined as a reduction in total red blood cell count or hemoglobin (Hb) and hematocrit (HT) concentration below the normal threshold or two standard deviation for age and sex ^1^^-^^3^ . Iron deficiency anemia (IDA) is the most common type of anemia in childhood and infancy, and is one of the major health problems across the world. This high prevalence is due to the basic role of iron in metabolism and nutrition. The body of neonate has about half a gram of iron, while this amount in adults is estimated to be about 5 grams. To compensate for this disparity, an average of 0.8 mg of iron should be absorbed in the first 15 years of our life. Addition to that portion of the iron which is needs for growth, some amount is needed to compensate for the iron loss during cell death. Therefore, in order to maintain a positive balance in childhood, 1 mg of iron must be absorbed into the body daily ^2^^, ^^4^ . According to the World Health Organization report, the prevalence of IDA in the world is about 30% (20-40%), but according to other reports, the prevalence of iron deficiency anemia in pregnant women, infants and children in developing countries is about 50-60%  ^5^^, ^^6^ . Even in industrialized countries, iron deficiency remains as a major cause of anemia in children^7^. According to studies conducted in Iran, the prevalence of this disease have been reported in students in Tehran as 3.8%^   8 ^, in children aged between 9 and 24 month, in Rafsanjan as 5.18%    9 , in Lorestan in children younger than 5 years as 26.5%^   10 ^, and in children aged 1-5 years in Kerman as 22.5%^11^. The causes of IDA include low birth weight, insufficient iron intake, increased iron need (following growth, congenital cyanotic heart disease), increased lysis (such as a condition in which hemolytic disease occurs), loss of Blood (following gastrointestinal bleeding, menstrual bleeding, epistaxis, etc.) and absorption impairment (malabsorption syndrome), celiac disease, chronic diarrhea after gastric surgery and in inflammatory bowel disease ^12^^-^^14^ . Also, because of the high growth rate in infancy and because the amount of iron in cow milk or maternal milk is about 1 mg, iron maintenance in infants is difficult^   15 ^. The most important symptom of iron deficiency is pallor, which is particularly visible on the conjunctiva and palms. In mild to moderate iron deficiency (hemoglobin 6-10 g / dl), irritability and pica (the tendency to eat abnormal objects such as ice and soil that are called pagophagia and geophagia, respectively) can appear. When the hemoglobin level reaches less than 5 g / dl, increased irritability and low appetite will become the hallmarks of the disease, and tachycardia and dilatation of the heart will appear, and often a systolic murmur will also be heard ^2^^,^^14^^,^^16^ . Iron deficiency can also affect neurological function and affect the level of attention, accuracy, alertness and learning ^17^^,^^18^. Also, iron with effect on lymphocytes and increased production of IL-2 and IL-6 increases the immunity against infections, which in case of iron deficiency, this property also decreases^14^^, ^^19^^,^^20^. Iron deficiency can also lead to thrombosis ^   21 ^. Alpha and beta thalassemia, anemia caused by chronic diseases and infections as well as lead poisoning can be found in the differential diagnosis of IDA  ^2^^, ^^22^ . The response of IDA to iron therapy is an important diagnostic and therapeutic aspect. The administration of oral ferrous salts is a cheap and successful treatment method. There is no evidence that the addition of rare metals, vitamins or other hematinic materials to simple ferrous salts significantly increase the response to treatment^   23 ^. Zinc is an important element that participates in the structure of enzymes, DNA and RNA, and is required for biochemical reactions in the body. Zinc deficiency is a common nutritional problem that is prevalent in developing countries, including Iran^   24 ^. Zinc deficiency is associated with reduced activity, attention, and impairment in motor development^   25 ^. Zinc deficiency also increases the severity of pneumonia, diarrhea and other infections^   26 ^. Since iron and zinc in the body, especially in terms of gastrointestinal absorption, have a fairly similar physiology, iron supplementation may interfere with intestinal absorption or plasma transfusion of zinc and other divalent elements in food, and leads to lack of these elements in the body  ^24^^, ^^27^ . Hence, it is expected that this effect will not be missed if both micronutrients, iron and zinc, are used together^   28 ^. According to the mentioned issues and the importance of having enough iron storage in the body to prevent its deficiency complications, it is very important to carry out studies on its deficiency treatment. Also, due to evidence of zinc reduction following iron supplementation, in this study, we investigated the effect of adding zinc compounds on accelerating the treatment of IDA in children suffering from this disease in our hospital.

## MATERIALS AND METHODS

 To conduct this interventional single blind study, ethical approval of our hospital and University Ethics Committee were obtained. Patients aged 6 months to 4 years old with mild to moderate IDA (hemoglobin level of more than 5 and less than 11, ferritin <12 or transferrin saturation <15%) who referred to our university hospital during 2016, and in the absence of exclusion criteria were enrolled into the study. Exclusion criteria were: Children with iron deficiency anemia who received any form of iron or zinc supplements in the last 6 months, children with any kind of chronic diseases, children with growth and weight disorders less than 3 percentile, children who had taken antiepileptic drugs for any reason, children who had any kind of blood product transfusion during the last 6 months, children with thalassemia major, children with severe IDA (hemoglobin level less than 5 ) or anemia who needed blood transfusion. After explaining all stages of the study to the patient's parents and receiving informed consent, demographic information, clinical symptoms and initial laboratory results were recorded in the pre-made questionnaire. Then, patients randomly were divided into two groups; group A (receiving 5 mg / kg/day of iron sulfate combined with 1 mg / kg/day of zinc sulfate) and group B (receiving iron sulfate alone at a dose of 5 mg / kg/day) for one month. After one month, laboratory parameters including CBC, TIBC and serum iron and ferritin level were re-checked and a history of possible clinical symptoms was re-taken. Finally, the data was processed using SPSS 15 (SPSS Inc., Chicago, Illinois, USA). Afterwards, the information about the treatment outcomes in the studied groups was analyzed by a statistician who was blinded to group allocation based on measures of central tendency criteria such as mean and standard deviation, using ANOVA and Paired T tests in Tables and charts. 

## Results

 A total of 88 patients were enrolled and divided into two groups: 48 patients (54.5%) in group A (zinc plus iron sulfate supplementation) and 40 patients (45.5%) in group B (iron sulfate supplementation). **Before treatment:** Two groups were matched with no difference according to the age and sex (Table-1). Also, before treatment symptom prevalence and lab data variables including hemoglobin, TIBC, ferritin and iron serum levels were not significantly different between the two groups (Tables 1 & 3). At the first visit, patients showed pallor (96.7%), irritability (95.6%), pica symptoms (18.9%) and poor appetite (93.3%), but none of the patients had symptoms of heart disease or pulmonary disease. 

**After treatment**: One month after treatment, in both A and B groups, pallor, irritability, pica and low appetite decreased dramatically (p<0.05) (Table-2). Also, study showed significant improvements according to the mean values of Hb, HCT, MCV, MCH, iron and ferritin serum level and TIBC after one month of treatment within each group (Table-4). However, at the same time, there was no significant difference between the two groups after one month of treatment. It means that there is no preference between the two types of treatment (Table-5).

## Discussion

 Iron deficiency anemia in childhood, especially in infancy, is very important because it can lead to irreversible developmental and cognitive deficits despite its easy way to prevent and treat. Supplementation with micronutrients can be easily considered as a therapeutic strategy for the prevention and treatment of IDA ^14^^, ^^29^^.^ The aim of this study was to compare the therapeutic effects of zinc plus iron supplementation and iron supplementation alone on improving the clinical and laboratory features of children with IDA referred to our hospital. In this study, a total number of 88 patients with IDA were studied, of whom 61 (69.3%) were boys and 27 (30.7%) were girls. Of 88 patients, 48 (54.5%) were in the A group and 40 (45.5%) were in the B group. The results of this study showed that the prevalence of symptoms such as pallor, irritability, pica and poor appetite decreased in both A and B groups, indicating that both therapeutic approaches relived symptoms dramatically and improved patients suffered from IDA and (Table-2). The study also revealed that one month after treatment the mean value of Hb, HCT, MCV, MCH, iron, ferritin and TIBC serum level significantly improved within each group compared to the pre-treatment amounts (P <0.05) (Table-4). However, the comparison of changes in laboratory variables between the two groups showed higher levels in group B (iron supplementation alone), but there was no statistically significant difference according to the type of treatment (p <0.05) (Table-5). In Munoz study, iron supplementation alone increased hemoglobin more than zinc plus iron combination, but both groups had a significantly greater increase than placebo group, which is indicative of positive effects of both approaches in the treatment of anemia^   30 ^. The mean increase in serum iron levels in the zinc plus iron recipient group was lower than the mean increase in the iron supplementation alone group, which Turgot also reported in his study^   31 ^. It should be noted that in the present study, the mean value of increased serum iron level was not significantly different between groups. Marjoline in his study reported that supplementation of zinc plus iron in comparison with iron alone was less effective in reducing anemia (20% vs. 38%), and no difference was observed in hemoglobin mean value and serum ferritin level between the groups which is in accordance with our study results^   15 ^. Lindsay ^   32 ^ and Hettiarachchi^   33 ^ also noted these results in their studies. In the Stanley Zlotkin study, cure rate in the group received iron supplement alone was also greater than those receiving zinc plus iron supplements (74.8% versus 62.9%)^   34 ^. Dijkhuizen also noted in his study that anemia reduction in the iron recipient group was greater than zinc plus iron recipient group (38% versus 20%), and he attributed this difference to the antagonistic effect of these micronutrients^   15 ^. In fact, this inhibitory effect of zinc on iron can be attributed to the competition of zinc with iron to attach to the same receptor in the intestinal lumen. These two micronutrients may also interact in each other's metabolism after absorption. Sometimes, this interference in absorption can even lead to iron deficiency itself, so that in the Donangelo study, after 6 weeks of taking zinc supplements, children who had enough iron storage had a 10% reduction in serum ferritin levels and this reduction was 25% in people who had marginal iron status^   35 ^. It means, long-term consumption of zinc can also cause iron deficiency in people who have enough iron storage. According to zinc supplementation, some studies reported that iron supplementation has no effect on zinc uptake and does not interfere with it  ^36^^,^^37^ . Although it has been stated in a study that iron significantly inhibits zinc absorption only in children who are not deficient in zinc, iron supplementation in people who have a sufficient supply of zinc decreases zinc level (9%), but in people with zinc deficiency it even increases to 25% and the researcher justifies this effect with the effect of these micronutrients on the divalent metal transporter-1 (DMT1)^   33 ^. In another study it is reported that zinc level increases in children who are treated with ferrous sulfate for 3 months, and this increase occurs when ferric iron is taken after 6 months^   38 ^. This result proves this claim that zinc deficiency in patients with iron deficiency is only treated with ferrous sulfate, and there is no need for zinc supplementation. According to the other studies, iron deficiency leads to structural disorders of the mucus and villi ^28^^,^^39^ , and this disorder can itself causes zinc deficiency due to malabsorption, which is the cause of zinc deficiency in people with iron deficiency. After the administration of ferrous sulfate to patients, according to the past hints, zinc levels will also rise and Zinc deficiency will be corrected. To prevent the effect of these micronutrients on each other, some studies have indicated that if their use together is necessary, zinc and iron should be used separately and with a certain interval to prevent interference with each other's absorption^29^^,^^37^. Some other studies have been mentioned that iron supplementation plus other factors such as vitamin A may have more effective role on the treatment of IDA than iron alone, although it is as controversial as zinc theory^40^^,^^41^. In addition to the effect of these different supplementations on anemia, some studies showed significant effects of both on increasing height, weight and head circumference mean values with no significant difference between different supplementations^42^^,^^43^, indicating this fact that the combination of iron and zinc is not more effective than iron alone on the growth and development of children. However, we could not verify that because we had such a short time period to work with and more research should be done to evaluate the effect of this combination.

**Table 1:** Demographic data and comparison of symptoms prevalence between the two groups before treatment (at first visit)

Symptom	Zinc plus Iron (N=49)	Iron alone (n=41)	P
**Mean age (months)**	25.7±2.24	22.25±1.74	Not sig
**Female patients**	14 (30%)	12 (32%)	Not sig
**Pallor**	48(98%)	39(95%)	Not sig
**Irritability**	45(92%)	41(100%)	Not sig
**Pica**	7(14%)	10(24%)	Not sig
**Low appetite**	45(92%)	39(95%)	Not sig

Quantitative data are presented as mean± standard deviation. Qualitative data are presented as number (%). P <0.05 is considered significant

**Table 2:** Comparison of symptom prevalence within each groups before and after treatment

Group	Symptom	Before treatment (At first visit) N (%)	After one month of treatment N (%)	P
**Zinc plus iron**	Pallor	48(98%)	3(6.3%)	sig
	Irritability	45(92%)	2(4.4%)	sig
	Pica	7(14%)	0(0%)	sig
	Low appetite	45(92%)	1(2.2%)	sig
**Iron alone**	Pallor	39(95%)	1(2.6%)	sig
	Irritability	41(100%)	3(7.3%)	sig
	Pica	10(24%)	0(0%)	sig
	Low appetite	39(95%)	3(7.7%)	sig

P <0.05 is considered significant

**Table 3:** Comparison of Lab data variables between the two groups **before treatment **(at first visit)

**Variable**	Zinc plus Iron Mean± SD	Iron alone Mean± SD	F	df	P
**Hb**	12.31±0.99	12.24±1.44	4.066	88	0.78 Not sig
**HCT**	37.28±2.97	37.99±8.72	1.981	88	0.594 Not sig
**MCV**	75.1±7.61	77.17±5.78	0.027	88	0.155 Not sig
**MCH**	22.25±1.74	25.68±2.23	1.325	88	0.305 Not sig
**Serum Iron**	36.59±15.69	34.065±14.52	0.021	88	0.434 Not sig
**Ferritin**	0.102±0.043	0.116±0.079	2.146	88	0.311 Not sig
TIBC	360.58±66.66	346.0±50.67	1.968	88	0.253 Not sig

Data are presented as mean± SD. SD; Standard Deviation, df; degrees of freedom. P< 0.05 is considered significant. Hb; hemoglobin, HTC; hematocrit, MCV; mean corpuscular volume, MCH; mean corpuscular hemoglobin, TIBC; total iron binding capacity

**Table 4:** Comparison of lab data variables within each group before and after treatment

Group	Variable	Before treatment (At first visit)	After one month of treatment	t	df	P
Zinc plus iron	Hb	12.31±0.99	12.62±0.73	-2.69	48	0.01
	HCT	37.28±2.97	38.93±5.53	-2.05	48	0.045
	MCV	75.1±7.61	77.34±4.06	-2.14	48	0.037
	MCH	25.25±1.74	25.62±1.66	-2.75	48	0.008
	Serum Iron	39.59±15.69	70.63±23.92	-10.128	48	0.0001
	Ferritin	0.102±0.043	0.219±0.082	-10.087	48	0.0001
	TIBC	360.58±66.66	330.61±53.06	3.156	48	0.003
Iron alone	HB	12.24±1.44	12.58±1.13	-2.36	40	0.023
	HCT	37.99±8.72	40.08±8.94	-2.16	40	0.037
	MCV	77.17±5.78	78.87±5.67	-3.28	40	0.002
	MCH	25.68±2.23	25.95±2.21	-0.99	40	0.327
	Serum Iron	34.065±14.52	70.75±28.14	-9.55	40	0.0001
	Ferritin	0.116±0.079	0.22±0.095	-6.24	40	0.0001
	TIBC	346.0±50.67	324.11±51.41	3.07	40	0.004

Data are presented as mean± SD. SD; Standard Deviation, df; degrees of freedom. P< 0.05 is considered significant.

**Table 5:** Comparison of lab data variables between the two groups after treatment

Variable	Zinc plus Iron Mean± SD	Iron alone Mean± SD	f	df	p
**Hb**	12.62±0.73	12.58±1.13	5.531	88	0.829
**HCT**	38.93±5.53	40.08±8.94	2.217	88	0.458
**MCV**	77.34±4.06	78.87±5.67	3.429	88	0.142
**MCH**	25.62±1.66	25.95±2.21	2.248	88	0.422
**Serum Iron**	70.63±23.92	70.75±28.14	1.365	88	0.982
**Ferritin**	0.219±0.082	0.222±0.095	1.881	88	0.876
**TIBC**	330.61±53.06	324.11±51.41	0.114	88	0.559

Data are presented as mean± SD. SD; Standard Deviation, df; degrees of freedom. P< 0.05 is considered significant. Hb; hemoglobin, HTC; hematocrit, MCV; mean corpuscular volume, MCH; mean corpuscular hemoglobin, TIBC; total iron binding capacity

## CONCLUSION

 The results of this study showed that taking iron alone and taking it in combination with zinc did not have a significant difference in the treatment of IDA, and the synchronic use of zinc and iron may increase some laboratory parameters such as Hb, but it is not significant. In general, the elimination of the health problems caused by anemia in children requires more comprehensive interventions with the aim of improving the status of iron and micronutrients in all parts of the society. Food enrichment, nutritional education and promoting culture and nutritional knowledge along with improving the quality of iron supplementation in childhood are interventions that can be used at the same time. Among the new strategies which currently are under consideration by health policy makers, we can point to the national program for the enrichment of baking flour with iron and folic acid, iron supplementation in high school girls, and the promotion of women's awareness, especially in deprived areas. One of the limitations of this study was the high cost of laboratory tests, as well as the limited number of cases who accepted to come back for evaluation over a longer period of treatment, resulting in difficulty to generalize the outcomes of the study to the general population. 

Further studies are suggested in larger communities and in longer time periods.

## References

[1] Rush D (2000). Nutrition and maternal mortality in the developing world. The American journal of clinical nutrition.

[2] Kliegman RM, Behrman RE, Jenson HB, Stanton BM (2007). Nelson Textbook of Pediatrics E-Book.

[3] Hess SY, Lönnerdal B, Hotz C, Rivera JA, Brown KH (2009). Recent advances in knowledge of zinc nutrition and human health. Food and nutrition bulletin.

[4] Anderson GJ, Frazer DM, McLaren GD (2009). Iron absorption and metabolism. Current opinion in gastroenterology.

[5] Silva DG, Priore SE, Franceschini SdC (2007). Risk factors for anemia in infants assisted by public health services: the importance of feeding practices and iron supplementation. Jornal de pediatria.

[6] Organization WH (2001). Iron deficiency anemia: assessment, prevention and control: a guide for programme managers.

[7] Sherry B, Mei Z, Yip R (2001). Continuation of the decline in prevalence of anemia in low-income infants and children in five states. Pediatrics.

[8] Shams S, Asheri H, Kianmehr A, Ziaee V, Koochakzadeh L, Monajemzadeh M (2010). The prevalence of iron deficiency anaemia in female medical students in Tehran. Singapore medical journal.

[9] Derakhshan S, Derakhshan R (2007). The prevalence of Iron deficiency anemia in 4-6 years old children of kindergardens at Rafsanjan City in 2006.

[10] Motlagh ME, Mardani M (1999). Anemia and Iron deficiency anemia in Lorestan province. Feyz Journal of Kashan University of Medical Sciences.

[11] Heidarnia A, Jalili Z, Dabiri S, Farahmandinia Z, Mohammad-Alizadeh S (1999). The prevalence of Iron deficiency anemia in 1-5 years old children referring to Kerman medical care and health centers in 1998. Journal of Kerman university of medical sciences.

[12] Poskitt EM (2003). Early history of iron deficiency. Br J Haematol.

[13] Kumar A, Rai AK, Basu S, Dash D, Singh JS (2008). Cord blood and breast milk iron status in maternal anemia. Pediatrics.

[14] Subramaniam G, Girish M (2015). Iron deficiency anemia in children. The Indian Journal of Pediatrics.

[15] Dijkhuizen MA, Wieringa FT, West CE, Martuti S (2001). Effects of iron and zinc supplementation in Indonesian infants on micronutrient status and growth. The Journal of nutrition.

[16] Brunner C, Wuillemin W (2010). Iron deficiency and iron deficiency anemia-symptoms and therapy. Therapeutische Umschau Revue therapeutique.

[17] Oski FA, Honig AS, Helu B, Howanitz P (1983). Effect of iron therapy on behavior performance in nonanemic, iron-deficient infants. Pediatrics.

[18] Akman M, Cebeci D, Okur V, Angin H, Abali O, Akman A (2004). The effects of iron deficiency on infants' developmental test performance. Acta Paediatrica.

[19] Ekiz C, Agaoglu L, Karakas Z, Gurel N, Yalcin I (2005). The effect of iron deficiency anemia on the function of the immune system. The Hematology Journal.

[20] Gera T, Sachdev H (2002). Effect of iron supplementation on incidence of infectious illness in children: systematic review. Bmj.

[21] Maguire JL, Parkin PC (2007). Association between iron-deficiency anemia and stroke in young children. Pediatrics.

[22] Lund EK, Wharf SG, Fairweather-Tait SJ, Johnson IT (1999). Oral ferrous sulfate supplements increase the free radical–generating capacity of feces from healthy volunteers. The American journal of clinical nutrition.

[23] Killip S, Bennett JM, Chambers MD (2007). Iron deficiency anemia. Am Fam Physician.

[24] Khoshfetrat M, Klantari N, Mohammadi Nasrabadi F, Rashidi A, Neyestani T, Abadi A (2008). Effects of Iron Supplementation With or Without Vitamin C on Oxidative Stress and Iron Status in Iron Deficient Female College Students. Iranian Journal of Endocrinology and Metabolism.

[25] Black MM (1998). Zinc deficiency and child development. The American journal of clinical nutrition.

[26] Brown KH, Peerson JM, Baker SK, Hess SY (2009). Preventive zinc supplementation among infants, preschoolers, and older prepubertal children. Food and nutrition bulletin.

[27] Fallahi a, Pouretemad h, Farhadi a (2009). Effect of iron and zinc supplementation on academic performance of primary school children. Journal of Shahrekord University of Medical Sciences.

[28] Gülsan M, Malbora B, Avcı Z, Bayraktar N, Bozkaya İ, Özbek N (2013). Effects of Zinc Sulfate Supplementation in Treatment of Iron Deficiency Anemia. Turkish Journal of Hematology.

[29] Walker CF, Kordas K, Stoltzfus RJ, Black RE (2005). Interactive effects of iron and zinc on biochemical and functional outcomes in supplementation trials. The American journal of clinical nutrition.

[30] Muñoz EC, Rosado JL, López P, Furr HC, Allen LH (2000). Iron and zinc supplementation improves indicators of vitamin A status of Mexican preschoolers. The American journal of clinical nutrition.

[31] Turgut S, Polat A, Inan M, Turgut G, Emmungil G, Bican M (2007). Interaction between anemia and blood levels of iron, zinc, copper, cadmium and lead in children. Indian journal of pediatrics.

[32] Allen LH, Rosado JL, Casterline JE, López P, Muñoz E, García OP (2000). Lack of hemoglobin response to iron supplementation in anemic Mexican preschoolers with multiple micronutrient deficiencies. The American journal of clinical nutrition.

[33] Hettiarachchi M, Liyanage C, Wickremasinghe R, Hilmers D, Abrams S (2008). The efficacy of micronutrient supplementation in reducing the prevalence of anemia and deficiencies of zinc and iron among adolescents in Sri Lanka. European journal of clinical nutrition.

[34] Zlotkin S, Arthur P, Schauer C, Antwi KY, Yeung G, Piekarz A (2003). Home-fortification with iron and zinc sprinkles or iron sprinkles alone successfully treats anemia in infants and young children. The Journal of nutrition.

[35] Donangelo CM, Woodhouse LR, King SM, Viteri FE, King JC (2002). Supplemental zinc lowers measures of iron status in young women with low iron reserves. The Journal of nutrition.

[36] Davidson L, Almgren A, Sandström B, Hurrell RF (1995). Zinc absorption in adult humans: the effect of iron fortification. British Journal of Nutrition.

[37] Fairweather-Tait SJ, Wharf SG, Fox TE (1995). Zinc absorption in infants fed iron-fortified weaning food. The American journal of clinical nutrition.

[38] Sözmen EY, Kavakli K, Çetinkaya B, Akçay YD, Yilmaz D, Aydinok Y (2003). Effects of iron (II) salts and iron (III) complexes on trace element status in children with iron-deficiency anemia. Biological trace element research.

[39] Ghosh S, Daga S, Kasthuri D, Misra R, Chuttani H (1972). Gastrointestinal function in iron deficiency states in children. American Journal of Diseases of Children.

[40] Kolsteren P, Rahman S, Hilderbrand K, Diniz A (1999). Treatment for iron deficiency anemia with a combined supplementation of iron, vitamin A and zinc in women of Dinajpur, Bangladesh. European journal of clinical nutrition.

[41] Murdiana A, Azis I, Saidin S, Jahari AB, Karyadi D (1988). Vitamin A-fortified monosodium glutamate and vitamin A status: a controlled field trial. The American journal of clinical nutrition.

[42] Alarcon K, Kolsteren PW, Prada AM, Chian AM, Velarde RE, Pecho IL (2004). Effects of separate delivery of zinc or zinc and vitamin A on hemoglobin response, growth, and diarrhea in young Peruvian children receiving iron therapy for anemia. The American journal of clinical nutrition.

[43] Lind T, Lönnerdal B, Stenlund H, Gamayanti IL, Ismail D, Seswandhana R (2004). A community-based randomized controlled trial of iron and zinc supplementation in Indonesian infants: effects on growth and development. The American journal of clinical nutrition.

